# Supplementary material for: A Plant-Produced Porcine Parvovirus 1-82 VP2 Subunit Vaccine Protects Pregnant Sows against Challenge with a Genetically Heterologous PPV1 Strain
Source: Vaccines (Basel). 2022 Dec 26;11(1):54. doi: 10.3390/vaccines11010054 (PMC9867127; doi:10.3390/vaccines11010054)
Supplement: Supplementary file 1 [file vaccines-11-00054-s001.zip › vaccines-2086015-SM.pdf]

vaccines

Article

A Plant-Produced Porcine Parvovirus 1-82 VP2 Subunit Vaccine Protects Pregnant Sows against Challenge with a Genetically Heterologous PPV1 Strain

Supplementary Table S1. Genetic diversity of VP2 amino acid (aa) sequences from different PPV1 strains. NADL-2, Kresse, Challenge, IDT, Tomau, T142, PVK2, and 27a are reference PPV1 strains, while strains 49, 25, 82, 13, 20, 21, 22, and 23 were identified in this study. The numbers in VP2 aa column are specified PPV1 VP2 aa sequence positions. The single letter codes represent the mutated amino acids, relative to the VP2 sequence of the NADL-2 reference strain; –, unmutated.

VP2 aa	PPV1 strain															
	NADL-2	Kresse	Challenge	IDT	Tomau	T142	PVK2	27a	49	25	82	13	20	21	22	23
28	G	–	-	-	-	-	-	-	S	S	-	-	-	-	-	-
45	T	S	S	S	S	S	S	S	S	S	S	S	S	S	S	S
143	Q	-	-	-	-	-	-	-	R	-	-	-	-	-	-	-
215	I	T	T	T	T	T	-	T	T	T	T	T	T	T	T	T
228	Q	-	-	-	-	-	-	E	-	-	E	-	-	-	-	-
270	D	-	-	-	-	-	-	-	-	-	N	-	-	-	-	-
320	I	-	-	T	T	-	-	-	-	-	-	-	T	T	T	-
378	D	G	G	G	G	G	G	G	G	G	G	G	G	G	G	G
383	H	Q	Q	-	Q	Q	Q	Q	Q	Q	Q	Q	Q	Q	Q	Q
407	K	-	-	N	N	-	-	-	R	R	-	R	-	-	-	-
414	A	-	-	-	-	-	-	S	T	T	S	-	-	-	-	-
419	E	-	-	-	-	-	-	Q	-	-	Q	-	-	-	-	-
436	S	P	P	P	P	P	P	T	T	T	T	P	P	P	P	P
549	T	-	-	-	-	-	-	-	A	-	-	-	-	-	-	-
565	R	K	K	K	K	K	K	K	K	K	K	K	K	K	K	K
570	Y	-	-	-	-	-	H	-	H	-	H	H	H	H	H	H
579	Y	-	-	-	-	-	-	-	-	-	-	-	-	S	S	-

A

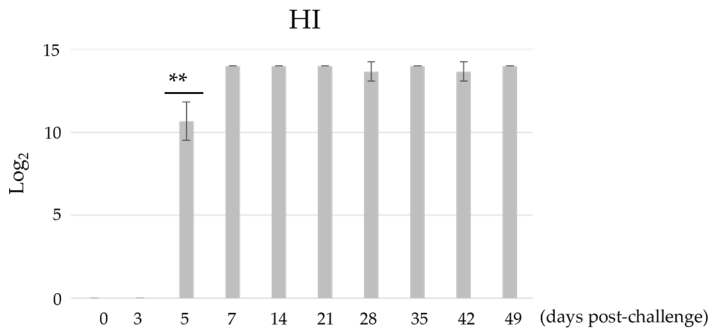

B

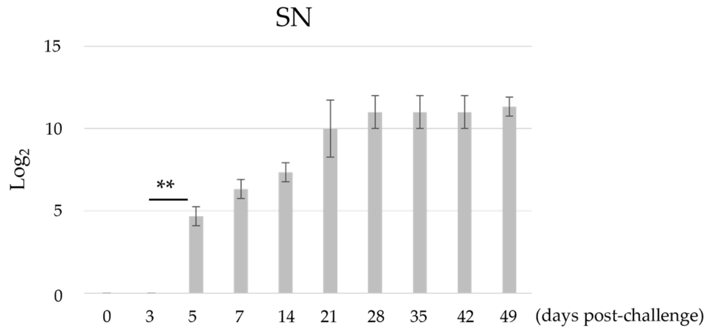

Figure S1. Hemagglutination inhibition (HI) and serum neutralizing (SN) antibody assays performed on samples taken on different days postchallenge, from three sows infected with the PPV1-190313 strain; $^{**}P < 0.01$.

Supplementary Table S2. Hemagglutination inhibition (HI) and serum neutralizing (SN) antibody values of vaccinated and control pregnant sows before and after PPV1-190313 challenge. The PPV1-82 VP2 vaccination was performed at 4 and 2 weeks before conception, and the sows were challenged with PPV1-190313 on gestation day 40. Blood samples were collected at 0, 7, 14, 21, 28, 35, and 49 days postchallenge (DPC). The HI test and PPV1-specific enzyme-linked immunosorbent assay (ELISA) were performed to qualify the amount of anti-PPV1 neutralizing antibodies.

Sows		HI (log_2)								
Group	Number	DPC								
		0	3	5	7	14	21	28	35	49
PPV1-82 VP2	1	13	13	13	14	14	14	14	14	14
	2	12	11	13	13	14	13	13	13	13
	3	13	12	13	13	14	14	13	12	12
PPV1-190313	1	< 2	< 2	10	14	14	14	14	13	13
Negative control	1	< 2	< 2	< 2	< 2	4	4	4	4	< 2

Sows		SN (log_2)								
Group	Number	DPC								
		0	3	5	7	14	21	28	35	49
PPV1-82 VP2	1	6	8	7	9	10	8	11	8	11
	2	7	5	7	8	12	12	10	11	9
	3	6	6	8	7	12	9	10	11	9
PPV1-190313	1	0	0	2	2	6	9	9	9	11
Negative control	1	0	0	0	0	0	0	0	0	0
